# Sida et Covid-19, deux maladies emblématiques de la santé mondiale

**DOI:** 10.48327/mtsimagazine.n1.2021.93

**Published:** 2021-04-28

**Authors:** S. Tchiombiano

**Affiliations:** Maîtresse de conférences associée dans le Département de science politique de l'Université Paris 1 Panthéon-Sorbonne, 12 Place du Panthéon, 75231 Paris et Coordinatrice du think tank « Santé mondiale 2030 », http://santemondiale2030.fr/; Cet article reprend certains éléments (rédigés par l'auteur) d'un chapitre de livre co-écrit avec Gilles Brucker, Yazdan Yazdanpanah et Michel Bourrelly, avec leurs accords respectifs: « VIH et Santé mondiale », in VIH, Katlama C. VIH, hépatites virales, santé sexuelle. EDP Sciences, 2020, pp. 821-839

**Keywords:** Santé mondiale, Sida, Covid-19, OMS, One Health, Multilatéralisme, Santé globale, Objectifs du Développement Durable, Epidémie, Pandémie, Politique internationale, Global health, AIDS, Covid-19, WHO, One Health, Multilateralism, Global health, Sustainable Development Goals, Epidemic, Pandemic, International policy

## Abstract

Alors qu'il existait depuis la fin des années 90, le concept de « Santé mondiale » fait aujourd'hui partie du langage courant des experts de la santé publique internationale, mais comment caractériser cette approche? Parce qu'elles transcendent les frontières, parce qu'elles appellent à des actions collectives et coordonnées au niveau mondial et parce qu'elles nécessitent une approche triplement transversale (multidisciplinaire, multi-acteurs et multisectorielle), les épidémies de sida et de Covid-19 illustrent parfaitement, chacune à sa façon, cette nouvelle approche. On peut considérer que la lutte contre le sida a été, d'une certaine façon, un laboratoire de la santé mondiale. En provoquant, avec d'autres, la réorganisation de l'architecture internationale de l'aide en santé, en stimulant l’émergence de nouveaux acteurs sur la scène internationale, en contribuant à l’éclatement des frontières et des disciplines, le sida a certainement été un accélérateur de cette nouvelle façon de penser les questions de santé. La crise de la Covid-19 transforme l'essai et oblige la communauté internationale à prendre conscience de ce nouvel impératif: nous n'avons pas d'autres choix que la santé mondiale, la coopération et la solidarité à l’échelle de la planète.

## Abstract (en)

While the concept of “Global Health” has existed since the late 1990s, it is now part of the everyday language of international public health experts, but how can this approach be characterized? Because they transcend borders, because they call for collective and coordinated actions at the global level, and because they require a tripartite approach (multidisciplinary, multi-actor and multisectoral), the AIDS and Covid-19 epidemics illustrate perfectly, each in its own way, this new approach. The fight against AIDS can be considered, in a way, as a laboratory for global health. By provoking, along with others, the reorganization of the international health aid architecture, by stimulating the emergence of new actors on the international scene, and by contributing to the breakdown of borders and disciplines, AIDS has certainly accelerated this new way of thinking about health issues. The Covid-19 crisis is converting the try and forcing the international community to become aware of this new imperative: we have no other choice than global health, cooperation and solidarity on a global scale.

Si le concept de Santé mondiale a pris une importance croissante depuis la fin des années 90, il est devenu incontournable avec la pandémie de Covid-19. La Santé mondiale est généralement définie comme « un domaine d’étude, de recherche et de pratique dédié à l'amélioration de l’état de santé et la recherche d'une équité en matière de santé, pour toutes les personnes, dans le monde entier » [1]. D'un point de vue plus opérationnel, cette nouvelle conception de la santé est liée à la mondialisation des échanges et des pratiques. Elle insiste à la fois sur la diffusion des problèmes de santé au-delà des frontières des pays qui les ont vus émerger, sur le caractère profondément transnational ou global des déterminants de santé et des facteurs de risque de ces maladies (qui peuvent être liées à la pollution, au climat, à l'alimentation, etc.) et sur l'incapacité des États à réguler seuls ces problèmes de santé. La Santé mondiale appelle à des solutions collectives, partagées et pensées au niveau global. L'avènement de la Santé mondiale serait donc à la fois révélateur d'un sentiment d'insécurité inédit, lié à de nouvelles menaces (mondiales ou potentiellement mondiales) et synonyme d'un nouvel élan collectif (mondial ou potentiellement mondial) pour répondre à ces nouvelles menaces. On utilise souvent l’épidémie de sida comme exemple pour illustrer cette nouvelle conception de la santé publique internationale. Certains vont même jusqu’à penser que la lutte contre le sida aurait inventé la Santé mondiale [2].

Les enjeux de santé mondiale se caractérisent en effet par trois dimensions complémentaires: (1) ils transcendent les frontières nationales, (2) ils appellent à des actions collectives et (3) ils imposent une approche transversale [1].

Sida et Covid-19 sont deux maladies infectieuses évidemment très différentes l'une de l'autre, par leurs natures, leurs modes de transmission, leurs histoires, par le fait aussi que l'une est aujourd'hui une maladie chronique, incurable, alors que l'autre ne l'est pas. Il ne s'agit pas ici de les comparer ou au contraire d’établir un rapprochement entre elles, mais juste de montrer de quelle façon elles ont, l'une et l'autre, contribué à l’émergence de l'approche Santé mondiale. Nous nous proposons donc de passer en revue les trois dimensions complémentaires de cette approche et de voir, pour chacune d'entre elles, dans quelle mesure ces deux maladies peuvent être considérées comme emblématiques de la Santé mondiale.

## Deux maladies qui transcendent les frontières

La première des trois dimensions de la Santé mondiale est donc le caractère profondément international des enjeux de santé qui la caractérisent. Au-delà de cette dispersion réelle des deux virus sur l'ensemble de la planète, trois facteurs ont certainement contribué à renforcer cette représentation des deux épidémies comme étant profondément mondiales (plus encore que les autres maladies transmissibles): les conditions spécifiques de leur découverte, les chocs qu'elles ont causés dans l'opinion publique internationale et les caractéristiques spécifiques de ces maladies (en particulier leurs modes de contamination).

### Deux virus profondément internationaux, dès leurs émergences

Pour l'infection à VIH, le fait que les premiers signalements aient été documentés en Californie en 1981, mais que le virus ait été découvert en France en 1983, par l’équipe de l'Institut Pasteur, puis la controverse avec les chercheurs américains qui revendiquaient également la découverte d'un virus dont on sait aujourd'hui qu'il vient d'Afrique centrale a certainement participé à la construction sociale de la maladie comme phénomène mondial. Les conditions initiales de diffusion de l’épidémie (par des travailleurs haïtiens venus travailler au Congo juste après l'indépendance [3]) vers le reste du monde sont évidemment emblématiques de l'interdépendance des pays et de l'accélération des échanges, mais elles n'ont évidemment rien à voir avec la vitesse de propagation de l’épidémie de Covid-19. En l'espace d'un seul mois, après les premiers cas déclarés officiellement en Chine (31 décembre 2019), 98 cas ont été recensés dans 18 pays différents. En dix semaines, 100 000 cas ont été documentés, sur tous les continents [4], démontrant l'interdépendance des pays et le rôle majeur des migrations dans la diffusion de l’épidémie au niveau mondial.

### Deux chocs inédits pour la communauté internationale

On peut considérer le sida comme la première grande alerte sanitaire mondiale de l'histoire. Jamais une maladie n'avait provoqué un tel choc, ni été l'objet d'une Assemblée générale extraordinaire des Nations Unies (UNGASS), en 2001. Le sida s'inscrit évidemment dans une histoire mondiale des maladies transmissibles et n'est pas, dans les faits, la première pandémie mondiale. Les spécialistes estiment par exemple que la peste ou la variole pouvaient également être considérées comme telles au Moyen Âge [5]. En revanche, ce qui différencie totalement le sida des maladies précédentes, c'est la prise de conscience de cette réalité.

Par la suite, bien d'autres maladies alerteront l'opinion publique internationale, comme la grippe aviaire H5N1 (1997), la grippe H1N1 (2009), le SRAS (2003), le chikungunya (2006) ou encore l'infection à Ebola (2014), mais aucune n'aura l'ampleur de la crise Covid-19. On retrouve avec la crise Covid-19 une sidération, puis un sentiment de gravité inédit face à l’émergence d'un virus inconnu (pourtant prévisible), amplifié cette fois, quarante ans plus tard, par l'importance de la médiatisation tous azimuts et des réseaux sociaux. Si le sida avait été lui aussi l'objet de nombreux fantasmes [6], cette nouvelle alerte sanitaire s'accompagne d'une importante « infodémie », mélangeant informations non vérifiées, fake news et théories du complot [7]. En contrepoint, le sentiment d'exaltation liée à la pandémie (autour du « Monde d'après », forcément durable, inclusif, solidaire) est finalement l'autre face du choc, profond et mondial, provoqué par la Covid-19.

### Deux maladies dont les modes de transmission relèvent de l'universel

Enfin, les caractéristiques propres de ces deux maladies infectieuses sont également à prendre en compte pour expliquer leur perception comme des maladies globales, universelles. Si le sida affecte des personnes dans la pleine force de l’âge alors que la Covid-19 impacte plus gravement les personnes âgées, les deux maladies ont la particularité de pouvoir être transmises par des personnes ne présentant aucun symptôme et de toucher potentiellement tout le monde. Leurs modes de transmission ont cela de commun qu'ils partagent une dimension profondément universelle: transmission sexuelle ou sanguine pour le sida, transmission orale, par aérosol, via la bouche, le nez ou les mains, pour la Covid-19. Ces modes de transmission qui touchent à l'intime et qui font de nous des êtres vivants (aimer, respirer, échanger, se toucher) renforcent encore cette perception de deux maladies profondément universelles. Ce caractère mondial de l’épidémie est bien sûr associé à l'idée d'une menace planétaire, mais il peut également être compris comme une source d'opportunité pour l'action collective.

## Deux maladies qui appellent à des actions collectives sur le plan international

La deuxième des trois caractéristiques des enjeux de santé mondiale est le fait qu'ils imposent une réponse globale, dépassent les capacités d'intervention des États, avec cette idée qu'une action coordonnée, collective, sera plus efficace que la somme des actions menées par chaque État au niveau national [8]. L'histoire internationale de ces deux maladies témoigne, de façon relativement différente, de cette dynamique collective, même si chacune d'entre elles a été considérée comme une crise sanitaire exceptionnelle, appelant une prise de conscience collective et une riposte planétaire, sur le plan institutionnel, financier et normatif.

### Dimension institutionnelle de la réponse mondiale

Avec le sida, une nouvelle architecture de l'aide en santé s'est progressivement mise en place, autour de programmes et d'agences mondiales totalement ou en partie dédiés au VIH: programme sida au sein de l'OMS (1986), partenariat ONUSIDA (1994) puis création d'initiatives publiques-privés avec le Fonds mondial de lutte contre le sida, la tuberculose et le paludisme (2002) et Unitaid (2006). Ces deux fonds multilatéraux inaugurent une nouvelle ère pour les mécanismes de gouvernance mondiale de la lutte contre la pauvreté [9]. Ils sont emblématiques de la mondialisation dans la mesure où ils marquent l'intrication du public et du privé, en dépassant les modèles classiques d'intervention centrés sur l'Etat, ouvrant leurs conseils d'administrations à d'autres acteurs (ONG, entreprises, fondations, associations de patients).

Renouvelant le modèle de financement de l'aide au développement [10], ces grandes initiatives seront sources d'inspiration pour des mécanismes du même type dans d'autres secteurs, comme l'environnement (Fonds vert pour le climat) ou l’éducation (Partenariat mondial pour l’éducation). Le sida, le paludisme et la tuberculose auront donc, à leur manière, inauguré une nouvelle forme de gouvernance mondiale de l'aide.

On peut dire que la coopération sanitaire interétatique a globalement été fragilisée par la crise de la Covid-19, exacerbant la compétition entre États (notamment pour l'accès aux vaccins) et les tensions géopolitiques (notamment entre la Chine et les États Unis). L'OMS a été particulièrement critiquée, pour n'avoir pas été suffisamment réactive et efficace, pour avoir tardé à déclarer la Covid-19 comme urgence de santé publique internationale (ce qu'elle ne fera que le 30 janvier 2020) puis comme « pandémie » (ce qu'elle ne fera que le 11 mars 2020) ou pour ne pas avoir été suffisamment ferme à l’égard de la Chine [11]. Des voix se sont élevées, appelant à la mise en place d'une nouvelle organisation internationale et à une reconfiguration de la gouvernance mondiale en santé. L'annonce de la suppression des financements américains à l'OMS en avril 2020 (finalement annulée suite à l’élection de Joe Biden) aura été l'acmé de cette remise en question.

Au-delà du système onusien, deux mécanismes publics-privés, associant théoriquement l'ensemble des acteurs (et non seulement les États) ont été mis en place: le dispositif « Act-A » (en anglais Access to Covid-19-19 tools Accelerator) et son sous-dispositif « Covax » (visant un accès mondial et équitable aux vaccins). S'il est encore trop tôt pour mesurer la pertinence et l'efficacité de ces deux innovations institutionnelles, elles incarnent en tous cas cette nouvelle perception du pouvoir politique, tout à fait emblématique de la Santé mondiale, selon laquelle il est nécessaire d'inclure le secteur privé (et théoriquement la société civile, ici particulièrement absente) au pilotage de l'action publique internationale.

### Dimension financière de la réponse mondiale

La lutte contre le sida a fait l'objet d'un financement international sans précédent, avec une augmentation moyenne des volumes financiers de 20% chaque année entre 2000 et 2013. Elle représente en 2020 quasiment un quart de la totalité des financements de l'aide en santé [12]. Si cette mobilisation financière a été particulièrement importante (et exemplaire), il est important de rappeler (1) que les dépenses liées au sida ont stagné, voire régressé depuis 2013 [12], (2) qu'elles sont très dépendantes du gouvernement américain qui représente à lui seul plus de la moitié du financement mondial [12] et (3) que, malgré les efforts, ces financements ne sont pas à la hauteur des besoins pour éliminer le sida comme menace pour la santé publique d'ici 2030 [13]. Au-delà de l'importance des volumes financiers récoltés grâce à cette dynamique mondiale, il est important de noter que le tiers des financements passe par des organisations multilatérales. La circulation des capitaux n'est plus seulement uniquement interétatique, elle ne dépend plus seulement de règles fixées par les États entre eux, elle est devenue globale au sens où elle se développe dans un cadre étendu à l’échelle du monde, et incluant des acteurs non étatiques, comme les fondations [14]. On retrouve avec la Covid-19 cette même logique d'un financement multi-bailleurs, au-delà de l'action bilatérale et des négociations entre États, pour répondre à des besoins de tests diagnostiques, de traitements, de vaccins qui sont universels, alors que les moyens financiers sont concentrés dans les pays les plus riches. Au-delà des efforts financiers faits par chaque État pour lutter contre l’épidémie au niveau national, des fonds doivent être mobilisés pour financer cette réponse globale.

Plusieurs sommets de donateurs ont été organisés, qu'ils soient sous l’égide de la Commission européenne (mai 2020) ou sous l’égide des Nations unies, du Canada et de la Jamaïque (mai à septembre 2020). Un appel à une « réponse multilatérale à grande échelle, coordonnée et globale, représentant au moins 10% du produit intérieur brut (PIB) mondial » [15] a été lancé à plusieurs reprises appelant les États à financer la lutte contre la Covid-19 au-delà de leurs besoins nationaux, au nom de la santé publique internationale et de la sécurité globale. Il est pourtant difficile d’évaluer l'ampleur réelle de la mobilisation collective car les promesses de dons collectées lors de ce type de sommets ne sont pas forcément suivies d'effets, qu'elles peuvent être uniquement liées à des réallocations de financements déjà alloués sur d'autres enjeux (sans être forcément additionnelles) et qu'il est difficile de faire la part des choses entre le financement direct de la lutte contre la Covid-19 et le financement de la lutte contre ses effets collatéraux. Si plusieurs initiatives tentent de mesurer la mobilisation financière internationale au fil de la riposte à la Covid-19 [16], il sera essentiel d'analyser avec attention et recul son ampleur réelle et ses modalités concrètes. On retrouve en tous cas dans les discours cet appel permanent à une approche partenariale, commune, à un panier commun et à la création de plateformes de mobilisation financière au-delà des États.

### Dimension normative de la réponse mondiale

Au-delà des dimensions institutionnelles et financières, les luttes contre ces deux maladies infectieuses sont également des moments de vérité de l'action publique internationale, dans sa capacité (ou non) à rassembler les forces vives autour d'une vision, d'objectifs, de principes et de stratégies de mise en œuvre communes (ou en tous cas coordonnées).

L'histoire de la lutte contre le sida est jalonnée de grandes initiatives (3 by 5, accès universel, 90-90-90, etc.) [17, 18 et 19]. S'ils étaient pour certains irréalistes, ces grands mots d'ordre, repris et diffusés par les organisations internationales, ont permis de lancer des dynamiques globales, de créer un agenda collectif et de rassembler l'ensemble des acteurs derrière des objectifs communs. Certaines règles ou certains principes sont par ailleurs devenus de véritables paradigmes de la lutte contre le sida: lutte contre la discrimination, approche par les droits, priorités données aux populations marginalisées, approche participative, égalité de genre, etc. Ces nouvelles normes structurent la lutte contre le sida, au sens où le respect de ces principes conditionne en général l'accès aux financements.

Enfin, au gré des avancées de la recherche et des résultats atteints, un certain nombre de stratégies opérationnelles ont été adoptées au niveau mondial, promues et déclinées en recommandations afin d’être mises en œuvre sur le terrain. On pourrait par exemple citer la succession de stratégies visant à promouvoir et à encadrer l'accès au dépistage (dépistage volontaire, dépistage ciblé vers des populations spécifiques, dépistage à l'initiative du soignant, stratégie « test and treat », etc.) et les controverses suscitées par cette question [20]. Ces politiques définies au niveau international sont censées ensuite être adaptées aux spécificités de l’épidémie dans chaque contexte. Elles sont dans les faits trop souvent mises en œuvre sur le terrain de manière standardisée, sans être forcément bien ajustées au contexte, aux réalités et aux dynamiques de chaque pays. Cette difficulté à trouver un juste milieu, entre homogénéisation et adaptation se retrouve largement dans la lutte contre la Covid-19. Ainsi, des mesures très fortes ont été prises, très rapidement par les autorités de certains pays africains pour lutter contre l’épidémie, en suivant l'exemple des pays précédemment touchés (fermeture des frontières, contrôles de température dans les aéroports, application des gestes barrières, confinement, distanciation physique), parfois avant même qu'un seul cas ne soit recensé sur le territoire national (comme au Lesotho, par exemple). Cela a certainement contribué à contenir l’épidémie mais ces mesures n’étaient pas toujours adaptées à la singularité des histoires et des contextes locaux, ou acceptables par les populations [21], et surtout, elles s'inspiraient relativement peu du savoir-faire spécifique des pays face à d'autres crises sanitaires (comme Ébola, par exemple), notamment en matière d'implication des acteurs communautaires. L'OMS doit évidemment jouer un rôle central dans la production et la diffusion de ces normes et recommandations internationales, dans un contexte marqué à la fois par l'urgence des décisions à prendre, par l'incertitude (face à ces épidémies nouvelles), par l’émergence de l’ « infodémie » et des fake news, par la complexité des rapports de pouvoir sur la scène internationale et par la récurrence des questionnements éthiques que posent l'action publique internationale [22].

## Deux maladies qui appellent une approche transversale

La dernière des trois caractéristiques des enjeux de santé mondiale réside dans le fait que ces enjeux doivent être compris selon une approche transversale de la santé. L'approche Santé mondiale postule non seulement que la santé est un droit, mais aussi qu'elle est un bien commun mondial. Cette vision globale de la santé prend en compte la complexité et l'interdépendance des différents déterminants de la santé, elle appelle donc à une approche multidisciplinaire, multi-acteurs et multisectorielle. Nous verrons que, tout comme la lutte contre le sida, la lutte contre la Covid-19 est tout à fait emblématique de ces trois aspects.

### Dépasser une approche biomédicale de la santé

La complexité des facteurs ou des déterminants de santé est l'une des caractéristiques fortes des deux épidémies. Si la transmission du VIH est en partie liée aux comportements individuels des personnes, la dynamique de l’épidémie dépend aussi en grande partie de facteurs socio-économiques (vulnérabilité économique, inégalités sociales, urbanisation), culturels (inégalités hommes-femmes, stigmatisation des minorités sexuelles, nombre de partenaires sexuels, diffusion de la circoncision, utilisation du préservatif, etc.) ou épidémiologiques (âge de l’épidémie, prévalence des infections sexuellement transmissibles, comorbidités, etc.). Lutter efficacement contre le sida oblige donc non seulement à une transdisciplinarité médicale autour du patient (infectiologie, dermatologie, médecine interne, etc.), à une transdisciplinarité au sein des sciences de la santé (médecine, santé publique, biologie, nutrition, pharmacie, épidémiologie, etc.), mais aussi, bien au-delà des sciences de la santé (anthropologie, sociologie, droit, économie, science politique. etc.).

Bien sûr, cette approche transdisciplinaire est pertinente pour bien d'autres problèmes de santé, mais l’épidémie de VIH, du fait de ses spécificités, a particulièrement mis en lumière son importance et son efficacité. On retrouve cette même nécessité d'inclure l'ensemble des disciplines dans la lutte contre la Covid-19 mais surtout, l’épidémie élargit encore le spectre des expertises à mobiliser, en pointant les liens de la santé des hommes avec l'environnement et avec la santé des animaux. Parce qu'elle met en lumière la cohabitation des hommes et des animaux, et la nécessité d'adopter une approche One Health [23], la réponse à la Covid-19 convie également autour de la table les climatologues, les zoologistes ou les vétérinaires.

### Coordonner une approche multi-acteurs

Au-delà des professionnels de la santé, la lutte contre le sida a révolutionné les savoir-faire et mobilisé de nouveaux acteurs de la société civile (association de personnes séropositives, acteurs communautaires, regroupement de populations particulièrement touchées ou vulnérables, organisations de défense des droits, etc.). Cette émergence de nouveaux acteurs s'est accompagnée d'une modification des pratiques: nouvelles dynamiques de concertation, protection des droits des patients, transparence des processus de décision, place des patients-experts, valorisation de l'expertise citoyenne. De nouveaux interlocuteurs ont progressivement émergé au niveau local comme au niveau international. Profondément ancrée dans une logique d'accès universel en santé, cette nouvelle dynamique insiste sur l'accès aux populations les plus vulnérables et marginalisées. Cette double volonté impose de nouvelles façons de faire, impliquant acteurs communautaires et secteurs privés. Des réseaux se sont mis en place progressivement: réseaux de recherche, réseaux associatifs, réseaux diplomatiques, et ont participé à la diffusion de nouvelles pratiques, de nouvelles idées ou de nouveaux savoirs. La lutte pour la gratuité des antirétroviraux a par exemple été un bel exemple de coalition entre les chercheurs, les associations de patients et les acteurs publics. L'environnement institutionnel de la lutte contre le sida a progressivement intégré ces nouveaux acteurs au nom d'une approche dite participative dans des mécanismes de gouvernance mondiale. S'il est indéniable que cette approche multi-acteurs a considérablement enrichi la lutte contre le sida au niveau mondial, elle l'a certainement également rendue plus complexe, chaque acteur conservant bien entendu ses stratégies, ses intérêts et sa façon de concevoir la lutte contre le sida.

Dans quelle mesure retrouve-t-on cette approche multi-acteurs dans la lutte contre la Covid-19? La crise a certainement confirmé ou amplifié le rôle des acteurs du secteur privé (place centrale de la Fondation Bill et Melinda Gates, rôle important des cabinets de conseil dans les dispositifs institutionnels, etc.), mais il semble évident que la société civile n'a pas eu, jusqu'alors, une place centrale dans la gouvernance de la Santé mondiale. Alors que la lutte contre le sida insistait sur les questions éthiques, la lutte contre les discriminations et la protection des droits individuels, la crise engendrée par la Covid-19 a plutôt été l'occasion de mesures menaçant les libertés individuelles [24]: fermeture de frontières, surveillance des citoyens (utilisation des drones en Chine, géolocalisation des personnes infectées), entraves à la liberté d'expression (Iran, Venezuela, Turquie, Honduras). La parole des citoyens a été relativement peu entendue [25], et si certaines associations ont joué un rôle absolument précieux au niveau local, elles ont été particulièrement absentes des instances de décision, au niveau national comme au niveau international [26].

Plusieurs éléments peuvent expliquer ces différences: 1) la lutte contre la Covid-19 ne s'inscrit pas (en tous cas pour le moment) dans des durées comparables à celles de la lutte contre le sida, 2) elle ne peut a priori, par nature, mobiliser des patients chroniques (elle pourrait en revanche impliquer des personnes guéries), 3) elle ne revêt pas la dimension intersectorielle de la lutte contre le sida qui était aussi, d'une certaine façon, une lutte pour les droits des minorités sexuelles, pour la liberté de choisir son orientation sexuelle. Quoi qu'il en soit, si l'on retrouve pour les deux épidémies, cette nécessité d'un logique multi-acteurs, propre aux enjeux de Santé mondiale, la dynamique peine à se mettre en place face à la Covid-19.

### Penser la santé en interdépendance avec les autres secteurs du développement

Le troisième aspect de cette transversalité spécifique de la Santé mondiale est son caractère transectoriel, avec cette idée que, face à une crise multifactorielle il est nécessaire de lutter en associant tous les secteurs: économie, éducation, environnement, etc. Cette approche est en parfaite cohérence avec la logique des Objectifs du développement durable (ODD), qui insiste sur l'existence de liens forts et interdépendants entre les différents secteurs. En fixant ces 17 objectifs communs, la communauté internationale semble s'inspirer en 2015 de l'approche multisectorielle que le sida explore et met en œuvre depuis plus de 30 ans. La création de l'ONUSIDA en 1994 (comme programme transversal à toutes les agences sectorielles des Nations unies) et la mise en place de programmes nationaux de lutte contre le sida directement rattachés au plus haut niveau de l’État (et non plus aux ministères de la santé) dans la grande majorité des pays africains témoignent de cette approche. Il y aurait beaucoup à dire sur l'efficacité des programmes qui ont été mis en place, et certainement beaucoup de leçons à tirer, mais il est en tous cas important de reconnaître le caractère précurseur de la lutte contre le sida en matière d'approche multisectorielle.

Cette même logique est à l’œuvre face à la Covid-19. Cette crise n'est pas seulement une crise sanitaire, elle est aussi une crise environnementale, sociale, politique, économique. Ainsi, 71 millions de personnes pourraient basculer dans l'extrême pauvreté (soit moins d’1,90 dollar US de revenu par jour), selon la Banque mondiale [27]. Pour être efficaces, les réponses à la Covid-19 doivent répondre à cette approche multisectorielle. Elles doivent non seulement s'attaquer aux facteurs sociaux, politiques, économiques qui rendent les populations vulnérables et freinent l'accès à la prévention, au dépistage, au traitement ou au vaccin, mais elles doivent aussi élargir le spectre et lutter contre les causes profondes de l’épidémie: l’émergence des zoonoses, l'anthropisation des espaces, la destruction de la biodiversité et le changement climatique.

## Conclusion

Par leur ampleur inédite, leur gravité et le choc qu'elles ont provoqué l'une et l'autre, sida et Covid-19 nous confrontent à des défis sanitaires, sociaux, économiques, politiques, sans précédents.

Non seulement le sida et la Covid-19 répondent aux trois caractéristiques des enjeux de Santé mondiale selon Jeffrey Koplan (transcender les frontières nationales, appeler à une action collective et imposer une approche transversale), mais ils sont, nous l'avons vu, totalement emblématiques de ces enjeux.

On peut considérer que la lutte contre le sida a été, d'une certaine façon, un laboratoire de la Santé mondiale. En provoquant, avec d'autres, la réorganisation de l'architecture internationale de l'aide, en stimulant l’émergence de nouveaux acteurs sur la scène internationale, en contribuant à l’éclatement des frontières et des disciplines, le sida a certainement été un accélérateur de cette nouvelle façon de penser les questions de santé mondiale, et peut-être même, plus largement, les questions de développement durable. La crise Covid-19 « transforme l'essai » et oblige la communauté internationale à prendre conscience de ce nouvel impératif: nous n'avons pas d'autres choix que la santé mondiale, la coopération et la solidarité à l’échelle du monde.

## Conflits d'intérêts

L'auteur ne déclare aucun conflit d'intérêt.
